# Semi-annual and annual mass drug administration of diethylcarbamazine and albendazole are equally effective regimens for eliminating lymphatic filariasis in Papua New Guinea

**DOI:** 10.1371/journal.pntd.0012979

**Published:** 2025-12-03

**Authors:** Michael C. Payne, Nelly Sanuku, Brooke E. Mancuso, Yao-Chieh Cheng, Gary J. Weil, Peter U. Fischer, Moses Laman, Leanne J. Robinson, Daniel J. Tisch, Christopher L. King

**Affiliations:** 1 Center for Global Health and Diseases, Department of Pathology, Case Western Reserve University, Cleveland, Ohio, United States of America; 2 Papua New Guinea Institute of Medical Research, Maprik, East Sepik Province, Papua New Guinea; 3 Infectious Diseases Division, Department of Medicine, Washington University School of Medicine, St. Louis, Missouri, United States of America; 4 Burnet Institute, Melbourne, Australia; 5 Department of Veterans Affairs, Cleveland, Ohio, United States of America; University of Passo Fundo: Universidade de Passo Fundo, BRAZIL

## Abstract

Annual mass drug administration (MDA) is currently recommended for the local elimination of lymphatic filariasis (LF). Modeling studies of LF transmission suggest that conducting MDA twice a year could accelerate LF elimination. To test this idea, we compared the effects of 3 rounds of yearly MDA and 5 rounds of semiannual MDA using diethylcarbamazine with albendazole on LF infection measures in Papua New Guinea (PNG) across 2 treatment areas with high LF prevalence. From 2013 to 2018, we conducted 4 annual community surveys at 4 sentinel sites in each treatment area. We sampled 2,854 people at the start and 2,746, 2,339, and 2,569 people at 13, 27, and 40 months, respectively, with a similar number of participants in each area. Yearly MDA reduced microfilariae (Mf) prevalence from 24.0% at baseline (95% confidence interval [CI] 22.9-26.1%) to zero (CI, 0-0.4%) at 40 months. Similarly, semiannual MDA lowered Mf prevalence from 23.3% (CI, 21.4-25.4%) to 0.3% (CI, 0.1-0.7%). The circulating filarial antigen (CFA) prevalence decreased from 46.4% at baseline (CI, 43.5-49.3%) to 29.5% (CI, 26.9-32.3%) after 40 months with yearly treatment and from 53.4% (CI, 51.0-55.9%) to 35.2% (CI, 32.7-37.8%) with semiannual MDA. Using a generalized estimating equation model that accounted for age, sex, bednet use, and sentinel site, we found no significant difference in the effectiveness of the two treatment approaches (p = 0.845 for Mf and p = 0.332 for CFA). Therefore, annual and semiannual MDA were equally effective in reducing LF prevalence in this high-endemic setting.

## Author summary

This study examines whether increasing the frequency of mass drug administration (MDA) can accelerate LF elimination. To test this hypothesis, we compared the effects of five rounds of semiannual MDA with three rounds of annual MDA, using diethylcarbamazine plus albendazole given to all eligible individuals in two areas of PNG with similarly high LF prevalence. Both treatment approaches, with identical community coverage, effectively reduced microfilaremia prevalence to below 1%, meeting the WHO threshold to indicate interruption of LF transmission. Annual MDA was just as effective as semiannual MDA, showing no additional benefit from more frequent treatment.

## Introduction

Lymphatic filariasis (LF) is a mosquito-borne infection caused by the filarial nematode parasites *Wuchereria bancrofti (Wb), Brugia malayi,* and *B. timori*. Adult worms live in the lymphatic vessels, impairing lymphatic function and leading to clinical consequences such as lymphedema, hydrocele, lymphangitis, and lymphadenitis [1]. Mosquitoes from the genera *Culex, Anopheles, Mansonia*, and *Aedes* transmit the parasites in various endemic areas.

The World Health Organization (WHO) estimates that over 657 million people in 39 countries worldwide are at risk for LF, with 15 million individuals disfigured and incapacitated by the disease [2]. To address this, the WHO initiated the Global Program to Eliminate Lymphatic Filariasis (GPELF), which aims to eradicate LF as a public health problem by 2020 [3]. The goal has since been revised to 2030 [4]. The elimination strategy involves community-wide mass drug administration (MDA) based on administrative units in endemic regions. Mass drug treatment consists of annual single-dose therapy with albendazole (ALB) combined with diethylcarbamazine (DEC) in countries not co-endemic for onchocerciasis. In areas co-endemic for onchocerciasis in sub-Saharan Africa, ALB is combined with ivermectin (IVM). In contrast, in regions where *Loa loa* is also prevalent and has high parasite loads, only ALB is administered [5]. Five annual rounds of MDA are recommended in most endemic countries; however, this has been difficult to sustain in countries with substandard rural health services. Some experts have proposed increasing the frequency of MDA to speed up LF elimination. A modeling study using LF infection data from West Africa and India predicted that semiannual treatment could accelerate the elimination process while offering significant cost savings [6]. This hypothesis was examined by comparing the impact of 5 semiannual MDA rounds to 3 annual MDA rounds in Indonesia and Liberia. The first study occurred on Flores Island in Indonesia, where *Wb.* and *B. timori* are endemic and mainly transmitted by *Anopheles* mosquitoes [7]. Both treatment regimens significantly reduced microfilaria (Mf) and circulating filarial antigen (CFA) prevalence; however, comparing the 2 regimens proved challenging due to significant differences in baseline LF prevalence between treatment areas. Two additional studies in different regions of Liberia, which also compared annual versus semiannual MDA (utilizing ivermectin and albendazole), found that 3 annual rounds were as effective as 5 semiannual rounds [8,9]. However, these LF endemic areas had prior MDA, and had moderate to low levels of LF prevalence.

Papua New Guinea differs from these other study sites by not having prior MDA, different Anopheles vectors, and higher LF prevalence. An estimated 6.4 million people in 14 of the country’s 22 provinces are at risk of infection. As of 2025, only 3 provinces, the implementation unit in PNG, have received MDA. At the time of study initiation in 2013, DEC plus ALB was recommended for yearly treatment for at least 5 years in PNG [10], however, no province had yet received MDA per WHO recommendations. In 2017, the WHO recommended a triple drug combination of IVM, DEC, and ALB or IDA [11]. This recommendation was adopted by PNG in 2018. By 2025, only 2 provinces had received MDA with IDA, but this did not include the province where this study was conducted. We aimed to examine whether 5 rounds of MDA given semi-annually over 3 years would reach LF elimination endpoints of <1% Mf and <2% CFA prevalence in the implementation areas faster than 3 annual rounds of MDA. Our findings show that 3 annual rounds of mass drug administration (MDA) with DEC and albendazole were as effective as 5 semiannual rounds of MDA in reducing LF prevalence.

## Methods

### Ethics statement

The study received approval from the PNG Institute for Medical Research IRB (# 1122), PNG Medical Research Advisory Council (MRAC #11.42), and University Hospitals Institutional Review Board in Cleveland, Ohio (#07-11-33). It was registered at ClinicalTrials.gov with the identifier NCT03268252. Adults provided written consent, and if they were unable to read or write, a literate community witness who was not involved in the study was present to sign with the participants’ assent. Parents or guardians gave consent for the children’s participation. Children aged 12 years and older were required to provide assent.

### Study area and population

This study was initiated in 2013 and completed in 2018. Included in this study were communities in the East Sepik and Sandaun Provinces in PNG. The study areas are in the foothills of the coastal mountain range with rugged topography, poor dirt roads, and subsistence farming of yams and taro in gardens in the rainforest. Cash crops are cocoa and vanilla. The study areas had no prior MDA for LF. Anti-helminth drugs such as albendazole and mebendazole are available without prescription in local pharmacies. However, these pharmacies are rarely used and distant from the study population. Long-lasting insecticide-treated bednets were introduced in this area in 2009, and new nets were distributed in 2012 and 2015.

**Fig 1:**
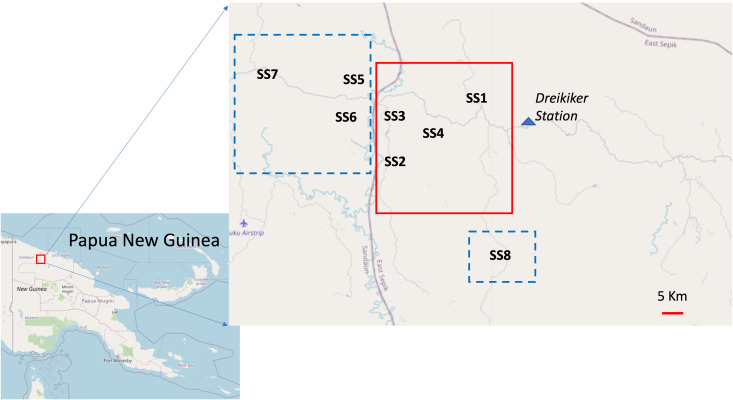
Sentinel sites located within areas treated with MDA semiannually (solid red line) or annually (dashed blue line). The Map is from OpenStreetMap (https://www.openstreetmap.org/).

The implementation unit in PNG is the province. At the time of this study, there were insufficient funds and inadequate infrastructure to treat the whole province, so MDA was distributed in parts of 3 Local Level Government (LLG) units in partnership with the East Sepik and Sandaun Provincial Health Authorities under the direction of the PNG National Department of Health (NoDH). The PNG NoDH supplied the drugs. Areas in Dreikikir Rural Local Level Government (LLG), East Sepik Province (ESP) received semi-annual MDA, while Palmai LLG in Nuku District of Sandaun Province and Gawanga LLG, ESP received annual MDA (Fig 1).

To assess the impact of MDA in the different LLGs, eight sentinel sites representing wards (the smallest administrative unit in PNG) that are comprised of 1–4 villages with a median population of ~1,000 (SS1–8, Fig 1) were selected for monitoring LF infection: 3 in areas that received annual MDA (Palmai LLG), and 1 outside the Palmai LLG (SS8, Gawanga LLG) and 4 in semi-annual treatment areas within Dreikikir Rural LLG. Several criteria were used to select sentinel sites: i) accessibility, ii) the ward had to have a total population of at least 500, iii) > 5 km from each other, and iv) local leaders were willing to participate. Only 3 of the 18 wards fit these criteria in Palmai LLG, so a fourth sentinel site was selected in another LLG (Gawanga LLG, SS8). For Dreikikir LLG, 9 of 33 wards fit these criteria and 4 were randomly selected from the 9.

Four annual cross-sectional surveys for LF were performed in each sentinel site: at baseline (before MDA), at 12 months, at approximately 27 months (range 24–29), and at approximately 40 months (range 37–42) after the first round of MDA. The households in each sentinel site were mapped and assigned numbers and randomly selected from a jar for sampling. All household members aged 5 years and older were asked to participate until approximately 400 individuals in each sentinel site agreed. The study recorded demographic information (gender, age, and bednet use), and participants were tested for Mf and CFA. This process was repeated for each cross-sectional survey at each sentinel site.

### Mass drug administration

Treatment comprised the WHO-recommended regimen of a single oral dose of diethylcarbamazine citrate (6 mg/kg body weight) with albendazole (a fixed dose of 400 mg). Annual MDA was provided at approximately 12–14 months intervals (baseline, 12, and 26 months) while semiannual MDA areas received 5 rounds over a similar period (baseline, 6, 13, 20 and 27 months) to persons ages 2 years and above per WHO guidelines. The number of people directly receiving antifilarial medications was observed by project staff. The compliance rates were estimated by dividing the number of people directly observed ingesting pills by the number of eligible residents in the census who were ≥ 5 years of age in treatment areas. The population aged 5 and older was used as the denominator because MDA was not distributed to younger children in some villages. The population was estimated from the 2011 national census. Compliance rates were monitored during the LF surveys.

### Field procedures

Field teams of trained nurses and field technicians met with community leaders. They held outdoor community education sessions to inform people about the health significance and biology of LF, the planned MDA program, and the importance of blood tests for monitoring the impact of MDA. Annual follow-up meetings were held to communicate preliminary results and to provide community members with opportunities to ask questions about the project.

Community liaison personnel mobilized village residents to participate in the study. Pregnant women and people with severe chronic illness or acute illness with fever were excluded from receiving MDA. We tested all individuals aged 5 years and older for LF. We reassessed eligibility for treatment each year. For example, pregnant women in year 1 were eligible for the study in later years if they were not pregnant. Survey teams enrolled participants in the late afternoon by obtaining oral informed consent from adults and recording demographic information. Enrollment of children required their assent and consent from at least 1 parent.

### Detection of Mf and filarial circulating antigen

Approximately 250 μL of blood by finger-stick was collected at night (between 8:30 and 12 PM) in an ethylenediaminetetraacetic acid (EDTA)-coated tube. Sixty microliters of blood were spread into 3 lines on a clean glass slide labeled with a unique barcode for each participant, dried for 2 days, dehemoglobinized for 3 minutes, air-dried, fixed with methanol for 1 minute, and stained with Giemsa for 15 minutes. Experienced laboratory technicians examined the stained slides for Mf by light microscopy. The remaining blood samples were centrifuged with plasma separated for antigen detection and stored at −20 C. Circulating *Wb.* antigen was detected with the Binax Now Filariasis Test (ICT) (Alere, Scarborough, ME) with whole blood at the time of blood collection for the baseline survey. The ICT tests were replaced by the better-performing semi-quantitative filarial test strip (FTS, Alere) for subsequent surveys (years 1–3). Test results were read 10 minutes after sample application according to the manufacturer’s instructions. The FTS results were scored as previously described [12] based on the intensity of the test (“T”) line. Test scores were recorded as follows: 0, no test line visible (negative test); 1, the test line is present but weaker than the procedural control line; 2, the test line is equal in intensity to the control line; or 3, the test line is stronger than the control line. Tests with no control line were considered invalid and repeated. The FTS detects a biomarker released by *Wb* adult worms and has a high sensitivity for detecting persons with microfilaremia. In clinical trials examining triple drug efficacy for LF in PNG and W. Africa, we found that all persons with microfilaremia (before and after treatment) had positive FTS results [13,14], and similar results have been reported from community field studies [15].

### Data management and statistical analysis

Data were captured using Blufones (Studio 5.0 II application) before synchronizing with the server using the ODK open-source software. Raw data were downloaded from the server as Excel files and subsequently uploaded into SPSS version 20 or SAS version 9.4 for analysis. Changes in Mf and CFA prevalence in each sentinel area pre- and post-treatment were analyzed using Fisher**’**s exact test. The geometric mean of Mf density was calculated using data from persons with microfilaremia. The effectiveness of MDA (annual vs semi-annual MDA) was compared over the 3 years of observation, using estimating equation models in SAS 9.4 (Carey, NC). The Genmod procedure was used with a binary link and independent correlation structure, controlling for 4 repeated observations and sentinel villages. Models fits were compared to alternative covariance structures (e.g., independent, exchangeable, AR1) using model diagnostics and the QIC statistic.

Sample size calculations were conducted as previously described [10]. In brief, a binomial power calculator with a significance level (α) of 0.05 (2-tailed) and statistical power of 0.80 was used in calculating sample size. The chosen sample sizes were intended to ensure sufficient confidence that observed prevalence rates would fall below specified thresholds, based on assumed true prevalence levels. At the sentinel site (village) level, the target sample size was approximately 400 participants per sentinel site—aligned with the minimum required to demonstrate, with 95% confidence and 80% power, that filarial antigen prevalence is below 2%, assuming an expected prevalence of 0.5%, consistent with the LF elimination threshold. The study aimed to enroll a total of at least 2,500 participants. To ensure the robustness of these estimates, the sample size analysis incorporated various contingency scenarios.

## Results

### Study population and treatment coverage

Between July and November 2013, participants were enrolled at eight sentinel sites in PNG (see Fig 1). The total number of people enrolled each year was 3175, 2735, 2360, and 2878 from the baseline through year 3 of follow-up, respectively (see Table 1). The number of females surveyed slightly exceeded that of males in the treatment areas. Other factors that could affect LF infection, such as age distribution (see Table 1) and the use of long-lasting insecticide-treated bed nets (LLIN), were similar between the 2 treatment arms (S1 Table). Bed nets were present in an average of 75% (range of 44% to 96% of sentinel sites) of households in sentinel sites receiving annual MDA and an average of 84% (range of 82% to 95%) of households receiving semiannual MDA.

**Table 1:** Prevalence of *Wb* microfilaremia and circulating filarial antigenemia before and after MDA in annual and semiannual intervention areas.

Semiannual MDA					Annual MDA			
	% Males	Median Age (IQR)	Mf Positive/N %Positive (95% CI)	CFA ^¶^ Positive/N %Positive (95% CI)	% Males	Median Age (IQR)	Mf Positive/N %Positive (95% CI)	CFA Positive/N %Positive (95% CI)
Pre-MDA	45	22 (13-37)	413/1772 23.3 (21.4-25.4)	893/1671 53.4 (51.0-55.9)	46	26 (16-39)	413/1720 24.0 (22.0-26.1.6)	549/1183 46.4 (43.5-49.3)
Year 1	48	19 (12-34)	71/1532 4.6 (3.6-5.8)	674/1520 44.3 (41.8-46.9)	48	18 (12-30)	30/1213 2.5 (1.6-3.5)	496/1226 40.5 (37.7-43.3)
Year 2	47	18 (13-32)	11/1019 1.1 (0.5-1.9)	427/1008 42.4 (39.3-45.5)	47	18 (12-32)	16/1328 1.2 (0.7-2.0)	430/1331 32.3 (29.9-35.0)
Year 3	49	18 (12-30)	4/1419 0.3 (0.1-0.7)	497/1411 35.2 (32.7-37.8)	50	15 (12-29)	0/888 0 (0.0-0.4)	342/1158 29.5 (26.9-32.3.0)

¶ Baseline circulating filarial antigen was measured with ICT card test and in subsequent years with the filarial test strip (FTS). IQR = interquartile range; CI = 95% confidence interval; MDA = mass drug administration; Mf = microfilaremia; CFA = circulating filarial antigen.

The proportion of individuals ingesting the drugs was evaluated at baseline and for each round of MDA for semiannual or annually treated sentinel sites (Table 2). The mean ingestion rates averaged over the 3-year study period were the same among the sentinel sites receiving semiannual treatment, 67.1% and annual treatment 68.1%.

**Table 2:** Directly observed antifilarial drug ingestion rates in semiannual and annual mass drug administration (MDA) areas.

MDA Round	Semiannual			Annual		
	Census	No. Treated	% Ingestion rate (95% CI)	Census	No. Treated	% Ingestion rate (95% CI)
MDA-1	3732	2697	72.3%	4170	2820	67.6%
MDA-1.5	4031	3041	75.4%			
MDA-2	3903	2474	63.4%	4210	3049	72.4%
MDA-2.5	3903	2624	67.2%			
MDA-3	3903	2431	62.3%	4210	2636	62.6%

### Impact of semiannual versus annual MDA on filarial infection

At baseline and during years 1–3, the prevalences of CFA and Mf were tracked in areas that received annual or semi-annual MDA (Table 1). The mean prevalences were calculated for all individuals in the 4 sentinel sites. Initially, the baseline Mf and CFA prevalences were the same between treatment areas (p = 0.769 and p = 0.884 chi-square for Mf and CFA respectively). The proportion of Mf-positive individuals varied from 9% to 35% in different sentinel sites (S1 Table).

One year after the first MDA, there was a 5–10-fold drop in Mf prevalence compared to baseline in both intervention arms. At 1 year, there was a trend toward a greater decline in Mf prevalence in the sentinel sites receiving a single round of MDA compared to semiannual sites after 2 rounds of MDA (Table 1). After 3 annual rounds of MDA with DA, no Mf-positive individuals were detected in any sentinel sites. In contrast, 4 Mf positive individuals were found in sentinel villages after 5 semi-annual rounds of MDA. The mean Mf prevalence at that time was only 0.3% (95% CI of 0.1%-0.7%) in semiannual sentinel sites. Two of 4 sentinel sites had Mf positive individuals, but all were <1% Mf (S1 Table). There was no significant difference in unadjusted mean prevalence between the 2 interventions at year 3 (p = 0.13, chi-square with Yates’ correction). Thus, both intervention sites achieved the WHO criteria of <1% Mf for the mean prevalence and in all the sentinel villages, suggesting interruption of LF transmission.

Changes in CFA prevalence followed those observed for Mf (Table 1). The CFA prevalences were equivalent at baseline between sentinel sites in the annual and semi-annual treatment areas. At years 2 and 3, the CFA prevalence was significantly lower in annual treatment groups in an unadjusted analysis year 2 (chi-square = 18.3, p < 0.0001) and year 3 (chi-square = 27.9, p < 0.0001).

**Fig 2:**
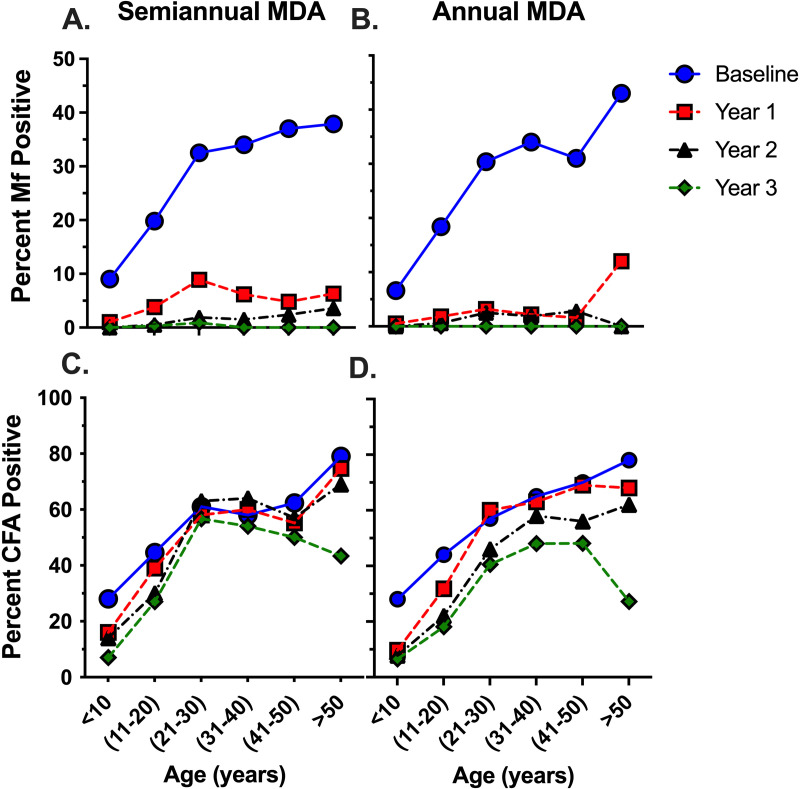
Changes in Mf (A and B) CFA (C and D) prevalence at baseline, 1, 2 and 3 years after starting MDA. Semiannual areas (A and C) received 5 rounds of MDA at approximately 6-month intervals, and annual MDA (B and D) was distributed yearly for 3 rounds of treatment. Of note, at the baseline, CFA used a different version of the antigen rapid test compared to years 1-3. Sample size for semiannual was age in years <10 = 332, 10-20 = 484, 21-30 = 338, 31-40 = 228, 41-50 = 178, > 50 = 124; annual was < 10 = 197, 10-20 = 353, 21-30 = 312, 31-40 = 235, 41-50 = 141, > 50 = 175.

**Fig 3:**
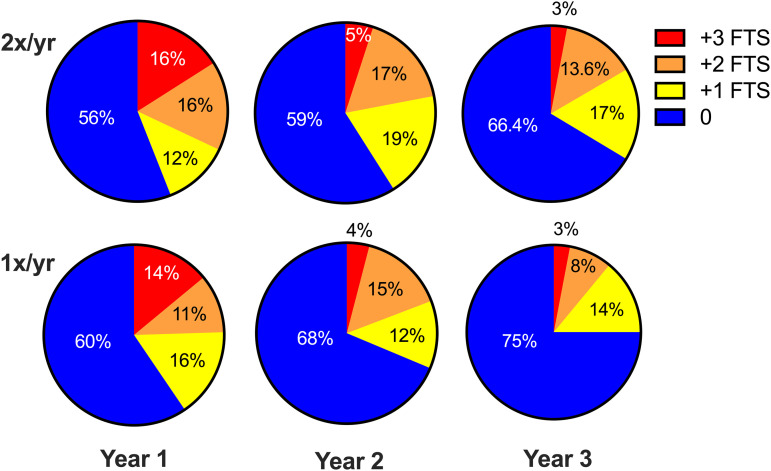
Changes in circulating filarial antigen (CFA) prevalence and levels at years 1, 2, and 3 following the start of mass drug administration, with treatment given semiannually (2x/yr) or annually (1x/yr). 0 indicates no detectable CFA, while 1 + , 2 + , and 3 + indicate low, moderate, and high CFA levels, respectively, as described in Methods. Baseline is not shown because a different rapid diagnostic test was used.

The impact of MDA on Mf reduction in the 2 treatment areas was observed in all age groups (Fig 2A and 2B). The decrease in CFA prevalence following MDA was observed in all age groups except for 21–30-year-old individuals in the semi-annual treatment zone (Fig 2). The intensity of filarial infection measured by semiquantitative CFA scores at years 1–3 also declined to similar degrees in the 2 treatment zones (Fig 3, ANOVA, p = 0.52). Note that baseline data CFA intensity is not shown because the rapid diagnostic test differed. The new, improved FTS only became available in year 1 of follow-up.

The effectiveness of MDA (annual vs semi-annual MDA) was compared using a generalized estimating equation model with a binary link and independent correlation structure, controlling for 4 repeated observations and sentinel village. Microfilaria and CFA prevalences were not significantly different between communities that had received annual vs. semi-annual MDA (p = 0.845 [Mf] and p = 0.332 [CFA]) or after adjusting for age, sex, bednet usage, and sentinel site (p = 0.728 [Mf] and p = 0.833 [CFA]). Rates of change were not statistically different between communities that had received annual vs. semi-annual MDA (p = 0.169 [Mf] and p = 0.134) or after adjusting for age, sex, bednet usage, and sentinel site (p = 0.092 [Mf] and p = 0.162).

## Discussion

A comprehensive national LF elimination program has recently been established in PNG. Only 3 of the 14 LF-endemic provinces have received MDA. At the time of this study, the PNG National Department of Health’s policy for LF elimination was 5 annual rounds of MDA with DEC plus Albendazole (DA). This recommendation partly resulted from local studies of anti-filarial treatments that included DEC alone, DEC plus ALB, and DEC plus IVM in PNG [16–18]. One study that administered 3 annual rounds of MDA with DEC plus ALB reduced Mf prevalence from 18.6% at baseline to 1.3% 1 year after the third round. While this showed that DEC plus ALB was highly effective in PNG, 3 annual rounds did not reduce the prevalence below 1% [18]. Subsequently, the PNG National Department of Health adopted WHO guidelines recommending 5 annual rounds of MDA at the provincial level for LF elimination. New Ireland Province became the first PNG province to undergo MDA with DA according to WHO guidelines. This island province received 3 annual rounds of MDA with DEC plus ALB from 2014 to 2016. It could not administer the final 2 rounds due to logistical challenges and a lack of funds. However, the mean prevalence of circulating filarial antigen (CFA) across the entire province, the evaluation unit, decreased from 17% before MDA to 0.3% after 3 rounds, with an average coverage of 78% (unpublished results). Still, some areas maintained LF prevalences above WHO targets of <2% CFA and <1% Mf positivity for the pre-transmission assessment survey (TAS). This underscores the need to shorten the duration of MDA programs and revise guidelines to focus on treating infected individuals after prevalence has been reduced to low levels.

The current study evaluated whether receiving 5 rounds of semiannual MDA with DEC plus ALB (DA) would lower LF prevalence more quickly than 3 annual rounds over 24 months. Final prevalence surveys occurred in all study areas about 12 months after the last MDA round. We selected a region in PNG with a high LF burden, with average Mf and CFA prevalences of 23% and 50%, respectively. The proportions of the total population aged 5 years and older who took the MDA medications were 68.1% and 67.5% overall in the annual and semiannual groups, respectively, meeting the WHO target of at least 65% coverage. Treatment was directly observed. Both treatment schedules significantly reduced Mf prevalence, with only 4 individuals remaining Mf positive (0.3%) after 5 rounds of semiannual MDA. No Mf-positive individuals were found in areas surveyed 1 year after 3 annual MDA rounds. Consequently, both MDA strategies brought Mf prevalence to well below 1%. There was no significant difference in how quickly or how much the LF infection parameters decreased between the annual and semiannual MDA over the 3-year period. Therefore, semiannual treatment offered no additional benefit.

Notably, there was no decrease in CFA prevalence among 21- to 30-year-old individuals in semi-annual group-treated areas. This age group is typically the most heavily infected, especially men, and their ingestion rates tend to be lower than those of other age groups because they are more likely to be traveling or working away from home and thus less available during MDA [19]. Circulating filarial antigen detects the presence of adult worms. Although DEC and albendazole are macrofilaricidal, they do not eliminate all adult worms, so in cases of heavy worm burdens, CFA can remain present even though levels generally decrease.

These results differ slightly from those reported from Madang Province (PNG), where 3 annual rounds of DA failed to reduce MF prevalence below 1% [18] even though that area had a lower baseline Mf prevalence (18.6%) and higher drug coverage at 78%. Annual MDA with DA was more effective in the current study, which may have been related to the recent introduction of long-lasting insecticide-treated bednets in the study areas in 2009. In villages near the current study sites, we studied the impact of LF transmission before and after the introduction of bednets [20]. Bednets significantly reduce the transmission of LF from 5 to 94% depending on the village [20]). It is possible that they reduced community transmission to the extent that administering drugs semiannually did not provide any additional benefit over annual administration in these study areas. Of note, in annual treatment sentinel sites, the Mf prevalence decreased by 91% after 1 round of MDA, although the treatment ingestion rate was 68.1%. Treatment coverage was estimated based on the number of people receiving directly observed treatment for the sentinel site and surrounding areas divided by the population based on the 2011 national census. The census and higher treatment coverage in the sentinel sites might have been inaccurate. Drug acceptability was high as people were directly observed taking the medications and rarely refused. This was the first MDA in the area, which otherwise receives little health care, and participants were willing to receive medication to feel better and reduce the burden of disease in the community. However, we did formally measure treatment acceptability in this study. Later studies formally studied drug acceptability in PNG [21], confirming this impression.

These results are consistent with similar published studies in Indonesia [7] and Liberia [8,9] and unpublished studies in Cote d’Ivoire, all of which found that five semiannual rounds of MDA provided no additional benefit in reducing LF burden compared to three rounds of annual MDA in different LF endemic areas worldwide with varying levels of LF endemicity, vectors, and drug combinations (DEC plus ALB in Indonesia and PNG or IVM plus ALB in Liberia and Cote d’Ivoire). After this MDA study was conducted, other research showed that a triple-drug treatment (ivermectin plus DEC and ALB, or IDA) given as a single co-administered dose was more effective at achieving sustained Mf clearance than either of the two-drug regimens [13,14,22,23]. As few as two annual rounds of MDA with IDA, when delivered with high coverage, may be enough to eliminate LF in many areas within PNG, especially in regions with high rates of insecticide-treated bednet use. In 2018, PNG adopted the new triple-drug regimen with one round of MDA following three rounds of DEC plus IVM in New Ireland Province (NIP). In 2021, NIP passed the first transmission assessment survey in children, making it the first province in PNG to do so [24]. In NIP, the evaluation unit was the entire province. In 2022, East New Britain Province completed two annual rounds of MDA with IDA, and West New Britain Province followed suit in 2024. Studies on the impact of IDA on LF infection parameters in these provinces are still pending.

There are study limitations and notable findings. The sentinel sites were often selected for logistical reasons, not randomly, and may not represent all the communities in the implementation unit. Mass drug administration (MDA) was not systematically administered throughout the whole implementation unit, but targeted the sentinel sites and surrounding areas. Of note, despite considerable variability of LF infection among the sentinel sites, the mean Mf and CFA prevalence were similar between sentinel sites in the semi-annual versus annual treatment areas. There was no prescreening of the sentinel sites, and this balance of prevalence was by chance.

In conclusion, this study has confirmed that mass drug administration (MDA) with diethylcarbamazine (DEC) plus albendazole (ALB) is highly effective for reducing LF infection parameters in PNG. However, semiannual MDA did not provide any additional benefit over annual treatment. Annual MDA works well when implemented adequately with high drug ingestion rates. The resources used for semiannual MDA should instead be allocated to improving the quality of annual MDA and supporting comprehensive monitoring and evaluation efforts to document success and identify persistent problem areas.

## Supporting information

S1 Table Lymphatic filariasis infection parameters stratified by sentinel site at baseline and during years 1 and 2 of MDA. Sentinel sites 1–4 received semiannual MDA twice a year, while sites 5–8 received annual MDA once a year. Year 3 samples were collected approximately one year after completing MDA.
(DOCX)

S1 Data Data. (XLSX)
