# Supplementary material for: Semi-annual and annual mass drug administration of diethylcarbamazine and albendazole are equally effective regimens for eliminating lymphatic filariasis in Papua New Guinea
Source: PLoS Negl Trop Dis. 2025 Dec 3;19(12):e0012979. doi: 10.1371/journal.pntd.0012979 (PMC12697938; doi:10.1371/journal.pntd.0012979)
Supplement: S1 Table — Sentinel sites 1–4 received semiannual MDA twice a year, while sites 5–8 received annual MDA once a year. Year 3 samples were collected approximately one year after completing MDA. (DOCX) [file pntd.0012979.s001.docx]

**S1 Table.** Lymphatic filariasis infection parameters stratified by sentinel site at baseline and years 1, 2 during the course of MDA. Sentinel sites 1-4 received semiannual MDA 2x/yr, and sentinel sites 5-8 received MDA annually (1x/yr). Year 3 samples were collected approximately 1 year following completion of MDA.
